# Troponins and Natriuretic Peptides in Cardio-Oncology Patients—Data From the ECoR Registry

**DOI:** 10.3389/fphar.2020.00740

**Published:** 2020-05-19

**Authors:** Lena Hinrichs, Simone Maria Mrotzek, Raluca-Ileana Mincu, Julia Pohl, Alina Röll, Lars Michel, Amir Abbas Mahabadi, Fadi Al-Rashid, Matthias Totzeck, Tienush Rassaf

**Affiliations:** Department of Cardiology and Vascular Medicine, West German Heart and Vascular Center, Medical Faculty, University Hospital Essen, Essen, Germany

**Keywords:** troponin, NT-proBNP, cancer, cardiotoxicity, cardiomyopathy, cardio-oncology

## Abstract

**Background:**

The long-term survival of cancer patients has significantly improved over the past years. Despite their therapeutic efficacy, various cancer therapies are associated with cardiotoxicity. Therefore, timely detection of cardiotoxic adverse events is crucial. However, the clinical assessment of myocardial damage caused by cancer therapy remains difficult.

**Methods:**

This retrospective study was performed to evaluate the diagnostic value of cardiac troponin I (cTnI) and N-terminal pro-B-type natriuretic peptide (NT-proBNP) for monitoring cancer therapy-induced cardiomyopathy. A total of 485 cancer patients referred to our cardio-oncology unit between July 2018 and January 2020 were selected from our *Essen Cardio-oncology Registry* (ECoR). We included patients with all types of cancer. Plasma concentrations of cTnI and NT-proBNP were measured by radioimmunoassay, and two-dimensional left ventricular ejection fraction (2D-LVEF), diastolic function, and global longitudinal strain (GLS) were measured by transthoracic echocardiography. In 116 patients, assessment was conducted before the induction of cancer therapy and during a short-term follow-up period; n = 42 of these were treated for malignant melanoma, and n = 42 with serial measurements were under treatment for breast cancer.

**Results:**

In cross-sectional data, elevated NT-proBNP was associated with reduced LVEF and pathological GLS in the total cohort. A total of 116 patients had serial LVEF and biomarker measurements, and changes in NT-proBNP and troponin correlated with changes in LVEF during follow-up investigations. Similar to the total cohort, a subgroup of patients treated for malignant melanoma showed a correlation between the change in cTnI and the change in LVEF. In a subgroup analysis of patients undergoing breast cancer therapy, a correlation between the change in NT-proBNP and the change in LVEF could be detected. Thirty patients presented with chemotherapy-induced cardiomyopathy, defined as a significant LVEF decrease (> 10%) to a value below 50%. The number of patients with increased cTnI and NT-proBNP was significantly higher in patients with chemotherapy-induced cardiomyopathy than in patients without cardiotoxicity. Patients with positive cTnI and NT-proBNP were more likely to have a history of coronary heart disease, atrial fibrillation, and arterial hypertension.

**Conclusion:**

Our data suggest that cardiac biomarkers play an important role in the detection of cancer therapy-induced cardiotoxicity. Larger systematic assessment in prospective cohorts is mandatory.

## Introduction

The development of antineoplastic treatments over the past decades has improved the long-term survival of cancer patients (Zamorano et al., 2016). Despite their therapeutic benefit, various cancer therapies are known for their cardiotoxicity (Lopez-Sendon et al., 2020). Conventional cytotoxic chemotherapy, radiotherapy, molecularly targeted inhibitors, antibodies directed against signal transduction pathways, and immune checkpoints can cause cardiovascular complications (Chang et al., 2017a; Chang et al., 2017b; Totzeck et al., 2017; Totzeck et al., 2018; Curigliano et al., 2019; Michel et al., 2019). Cardiotoxic effects can lead to acute and chronic heart failure, myocarditis, thromboembolic events, arrhythmias, and coronary artery disease, contributing to increased cardiovascular mortality in cancer survivors (Chang et al., 2017a; Chang et al., 2017b). Early identification of subclinical cancer therapy-related cardiotoxicity could lead to an early initialization of cardioprotective strategies, reduce cardiovascular mortality, and prevent the interruption of mandatory antineoplastic therapy (Zamorano, 2016; Totzeck et al., 2019a; Totzeck et al., 2019b; Curigliano et al., 2020). The early detection of cancer therapy-related myocardial dysfunction is one of the major challenges in the field of cardio-oncology (Rassaf and Totzeck, 2018; Cardinale et al., 2019). Chemotherapy-induced cardiomyopathy is defined as a reduction in left ventricular ejection fraction (LVEF) of more than 10% to a value below 50% (Zamorano, 2016), but due to delayed manifest changes of LVEF, echocardiography may lack sufficient sensitivity to detect early subclinical cancer therapy-related cardiotoxicity (Santoro et al., 2017; Skubitz et al., 2017). Cardiac biomarkers play an important role in the detection and monitoring of acute and chronic myocardial injury. Despite their wide and fast availability, as well as accuracy and reproducibility, the routine use of cardiac biomarkers in tumor patients for the detection of chemotherapy-induced cardiotoxicity has not yet been established.

### Troponin

Cardiac troponins are structural proteins of the contractile apparatus and are expressed almost exclusively in myocardial cells. Only a small proportion of troponins are expressed in the cytoplasm of myocytes. Integrated in the thin filament of the sarcomere of striated muscle, cardiac troponins are involved in electromechanical coupling (Giannitsis and Katus, 2013). They consist of three subunits: troponin C, I, and T. The detection of both cardiac troponin I (cTnI) and T (cTnT) plasma concentrations is an established marker for the detection of acute and chronic myocardial damage with high sensitivity and high informative value regarding diagnosis and prognosis (Rubini Gimenez et al., 2014; Thygesen et al., 2018). The main pathomechanism of troponin release is myocardial ischemia leading to myocardial necrosis (Katus et al., 1989). The use of cardiac troponin for the detection of acute myocardial infarction has been established in guidelines for years (Thygesen et al., 2018). Population studies suggest that patients with an elevated plasma concentration of troponins show increased cardiovascular mortality and are at higher risk for cardiac adverse events (Hsieh et al., 2009). However, the development and time course of troponin elevation caused by antineoplastic treatment are more variable and less clearly investigated than those caused by conventional cardiovascular disease (Cardinale et al., 2000; Cardinale et al., 2015; Cardinale et al., 2017; Lopez-Sendon et al., 2020).

### NT-proBNP

Brain natriuretic peptides (BNPs) are hormones released by ventricular cardiomyocytes in response to increased filling pressures and improve myocardial performance due to vasorelaxation and natriuresis (Weber et al., 2006). The prohormone of BNP, NT-proBNP, has a comparably longer half-life in plasma (Weber et al., 2006). The elevation of serum levels of NT-proBNP is an established biomarker for the diagnosis of heart failure and an independent risk factor for cardiovascular events (Maisel et al., 2001; Ponikowski et al., 2016). The reduction of LVEF is the most common cardiotoxic event from antineoplastic therapy (Tan and Lyon, 2018). Interestingly, recent clinical trials and observational studies addressing NT-proBNP in cardiotoxicity had inconsistent results regarding the correlation of elevated NT-proBNP and cancer therapy-related heart failure (Sawaya et al., 2011; Sawaya et al., 2012; Wang et al., 2016).

### Aim of the Study

To date, there is inconsistent evidence for conventional biomarkers as markers of cardiotoxicity in cancer patients. The aim of this retrospective investigation was to determine whether levels and changes in cTnI and NT-proBNP may correlate with the occurrence of LVEF reduction in cancer patients and to evaluate potential associations between biomarker levels and clinical data.

## Methods

### Study Population

This retrospective investigation included n = 485 patients who presented to our cardio-oncology unit between July 2018 and January 2020 and were included in our ‘*Essen Cardio-oncology Registry*' (ECoR). The inclusion criteria were as follows: cancer patients ≥ 18 years with a histologically confirmed diagnosis with Eastern Cooperative Oncology Group (ECOG) status I–II and planned or initiated cardiotoxic antitumor therapy (chemotherapy, immune therapy, radiation). Bedridden patients with ECOG III-IV were excluded. In n = 116 patients, assessment was conducted before cancer therapy and during a short-term follow-up period (1–6 months after the start of antineoplastic treatment), while in the remaining patients, data from only one assessment were available. The choice of follow-up termination depended on the antineoplastic treatment and clinical indication and was left to the treating physician. As part of the presentation to our cardio-oncology unit, patients were clinically examined and interviewed to determine signs of heart failure and myocardial ischemia. On the basis of symptom burden, dyspnea and angina pectoris were classified according to the *Canadian Cardiovascular Society* (CCS) and the *New York Heart Association* (NYHA) classifications (Ponikowski et al., 2016; Thygesen et al., 2018). Details about comorbidities, blood pressure, heart rate, and oncological disease and treatment were obtained from medical records. The ECoR database was approved by the institutional ethics committee of the University of Duisburg-Essen (Essen, Germany, 19-8632-BO) and conformed to the principles of the Declaration of Helsinki.

### Transthoracic Echocardiography

Echocardiographic examinations were performed using an EPIQ 7 (Philips Healthcare, Germany) ultrasound machine. Echocardiographic analysis of cardiac structure and function was performed in accordance with the recommendations of the American Society of Echocardiography (ASE) and European Association of Cardiovascular Imaging (EACVI) (Galderisi et al., 2017; Mitchell et al., 2019). Left ventricular end-diastolic and end-systolic volumes were assessed in the apical 4- and 2-chamber view, and 2D-LVEF was evaluated using the biplane method according to Simpson's rule (Mitchell et al., 2019). Global longitudinal strain was measured by *post hoc* analysis with the speckle-tracking method using Qlab software (Philips Healthcare, Germany). The endocardial borders of the left ventricle were traced in 4-, 2-, and 3-chamber views, and Qlab software automatically tracked endocardial borders throughout the cardiac cycles. The software calculated myocardial strain by using the speckle-tracking method, and myocardial strain was defined as the change in an object's dimension in comparison to the object's original dimension. Moreover, diastolic dysfunction was assessed according to current recommendations (Galderisi et al., 2017; Mitchell et al., 2019). Therefore, the investigation of pulsed-wave Doppler of transmitral flow and myocardial velocities of the lateral mitral annulus during early diastole (E′) by tissue Doppler were obtained in the apical four-chamber view. Subsequently, the E/E′ ratio was calculated. Chemotherapy-induced cardiomyopathy was defined in accordance with the current recommendations as a reduction in left ventricular function of more than 10% to a value below 50% (Zamorano, 2016). The examination was performed by two qualified, experienced echocardiographers with low inter- and intraobserver variability.

### Biomarker

Plasma concentrations of cTnI and NT-proBNP were measured by radioimmunoassay (ADVIA Centaur TnI-Ultra Assay, Siemens; IMMULITE 2000 NT-proBNP, Siemens). cTnI was considered positive for values >6 ng/L and significantly elevated at a level >40 ng/L (99th percentile). NT-proBNP was considered positive at a level of >125 pg/ml.

### Statistical Analysis

All data were pseudonymized (Microsoft GmbH). Statistical analysis was performed using GraphPad Prism software version 6.0 and IBM SPSS Statistics. The data were checked for normal distribution using the Kolmogorov-Smirnov test. Troponin and NT-proBNP values showed a non-normal distribution. Differences in proportions of dichotomous values between groups were compared with univariate and multivariate binary logistic regression analyses. Moreover, Spearman correlation analysis was performed to investigate the association between LVEF and biomarkers between baseline and follow-up investigations. Furthermore, a sensitivity and specificity analysis was performed. A *p* value < 0.05 was considered statistically significant. Continuous variables are shown as the mean ± the standard deviation (SD).

## Results

### Characteristics of the Study Population

A total of n = 485 patients were included from our ECoR cardio-oncology database, and n = 116 patients had serial measurements for LVEF and biomarkers. Characteristics of the study population are provided in **Tables 1** and **2**.

**Table 1:** Characteristics of the study population.

	cTnI <6 ng/L (n = 387)	cTnI 6–40 ng/L (n = 63)	cTnI > 40 ng/L (n = 35)	NT-proBNP >125 pg/ml (n = 159)	Total cohort (n = 485)
**Age (years)**	61 ± 15	62 ± 12	62 ± 18	63 ± 15	62 ± 15
**Women/men**	202/185 (52/48)	22/41 (35/65)	14/21 (40/60)	68/91 (43/57)	245/240 (51/49)
**Systolic blood pressure (mmHg)**	122 ± 21	128 ± 20	130 ± 20	129 ± 21	130 ± 20
**Heart rate (bpm)**	78 ± 18	78 ± 17	85 ± 17	78 ± 17	79 ± 18
**Cardiotoxicity**	18 (5)	8 (13)	4 (11)	21 (13)	30 (6)
**Comorbidities**					
Coronary artery disease	58 (15)	15 (24)	10 (29)	40 (25)	83 (17)
Smoking	44 (11)	14 (22)	6 (17)	33 (21)	64 (13)
Atrial fibrillation	38 (10)	14 (22)	7 (20)	29 (18)	59 (12)
Hypertension	156 (40)	33 (52)	17 (49)	60 (38)	206 (42)
COPD	21 (5)	2 (3)	1 (3)	11 (7)	24 (5)
Diabetes	25 (6)	13 (21)	3 (9)	23 (14)	41 (8)
Hypercholesterolemia	41 (11)	12 (19)	5 (14)	30 (19)	58 (129)
Stroke	13 (3)	4 (6)	2 (6)	9 (6)	19 (4)
High-grade valvular disease	5 (1)	1 (2)	3 (9)	6 (4)	9 (2)
**Echocardiographic findings**					
LVEF					
<40%	24 (6)	7 (11)	11 (31)	24 (15)	42 (9)
40–50%	39 (10)	20 (32)	15 (43)	62 (39)	74 (15)
>50%	334 (86)	25 (40)	10 (29)	73 (46)	369 (76)
Diastolic dysfunction					
I°	46 (12)	9 (14)	4 (11)	21 (13)	59 (12)
II°	22 (6)	8 (13)	3 (9)	16 (10)	33 (7)
III°	2 (1)	1 (2)	3 (9)	5 (3)	6 (1)
Global longitudinal strain					
<−20	47 (12)	20 (32)	7 (20)	43 (27)	74 (15)
>−20	128 (33)	8 (13)	1 (3)	16 (10)	136 (28)
**Symptoms**					
Dyspnea (NYHA stage)					
NYHA I	107 (28)	7 (11)	3 (9)	27 (17)	117 (24)
NYHA II	151 (39)	23 (37)	13 (37)	67 (42)	187 (39)
NYHA III	29 (7)	10 (16)	13 (37)	39 (25)	52 (11)
NYHA IV	3 (1)	0 (0)	2 (6)	4 (3)	5 (1)
Angina pectoris (CCS stage)					
CCS I	21 (5)	7 (11)	3 (9)	17 (11)	31 (6)
CCS II	5 (1)	1 (2)	2 (6)	4 (3)	8 (2)
CCS III	0 (0)	0 (0)	5 (14)	5 (3)	5 (1)

Values are presented as the mean ± the standard deviation or the number of patients (percent).

bpm, beats per minute; cTnI, cardiac troponin I; CCS, Canadian Cardiovascular Society; COPD, chronic obstructive pulmonary disease; LVEF, left ventricular ejection fraction; n, number; NT-proBNP, N-terminal pro B-type natriuretic peptide; NYHA, New York Heart Association.

**Table 2:** Oncological diseases and antineoplastic treatments.

	cTnI <6 ng/L (n=387)	cTnI 6–40 ng/L (n=63)	cTnI > 40 ng/L (n=35)	NT-proBNP > 125 pg/ml (n=159)	Total cohort (n=485)
**Oncological disease**					
Breast cancer	104 (27)	8 (13)	6 (17)	35 (22)	118 (24)
Malignant melanoma	116 (30)	11 (17)	3 (9)	22 (14)	130 (27)
Multiple myeloma	23 (6)	6 (10)	3 (9)	13 (8)	32 (7)
Lymphoma	28 (7)	2 (3)	6 (17)	14 (9)	36 (7)
Leukemia	39 (10)	8 (13)	9 (26)	24 (15)	56 (12)
Small-cell lung cancer	13 (3)	1 (2)	1 (3)	5 (3)	15 (3)
Merkel cell carcinoma	8 (2)	0 (0)	4 (11)	6 (4)	12 (2)
Gastrointestinal cancer	11 (3)	3 (5)	0 (0)	12 (8)	14 (3)
**Antineoplastic treatment**					
Conventional chemotherapy (total)	243 (63)	38 (60)	29 (83)	117 (74)	310 (64)
Anthracycline	87 (22)	13 (21)	14 (40)	51 (32)	114 (24)
Alkylation	148 (38)	27 (43)	20 (57)	59 (37)	195 (40)
Taxane	61 (16)	5 (8)	13 (37)	11 (7)	79 (16)
Pyrimidine antagonist	11 (3)	4 (6)	3 (9)	11 (7)	18 (4)
Purine antagonist	10 (3)	4 (6)	1 (3)	5 (3)	15 (3)
Proteasome inhibitor	4 (1)	0 (0)	3 (9)	4 (3)	7 (1)
Tyrosine kinase inhibitor	52 (13)	9 (14)	1 (3)	17 (11)	62 (13)
HER2/neu inhibitor	24 (6)	3 (5)	4 (11)	15 (9)	31 (6)
PD1 inhibitor	90 (23)	9 (14)	4 (11)	25 (16)	103 (21)
BRAF inhibitor	17 (4)	3 (5)	1 (3)	4 (3)	21 (4)
MEK inhibitor	21 (5)	2 (3)	2(6)	5 (3)	25 (5)
Radiation	89 (23)	13 (21)	5 (14)	39 (25)	107 (22)
Surgery	149 (39)	22 (35)	10 (29)	58 (36)	181 (37)

Values are presented as the number of patients (percent)

BRAF, B-rapidly growing fibrosarcoma; cTnI, cardiac troponin I; HER2, human epidermal growth factor receptor 2; MEK, mitogen-activated protein kinase; NT-proBNP, N-terminal pro-B-type natriuretic peptide; PD1, programmed cell death protein 1.

### Associations of Patient Characteristics With Positive Troponin or NT-proBNP

Univariate and bivariate analyses were performed to detect potential associations between the population characteristics and biomarkers, and the results are presented in **Tables 3** and **4**. In univariate analysis, positive cTnI (OR, 0.50 [0.31 to 0.80], *p* = 0.004) and NT-proBNP values (OR, 0.64 [0.44 to 0.93], *p* = 0.021) were more frequently observed in male patients. Age, systolic blood pressure, and heart rate did not show any detectable differences between patients with and patients without cTnI and NT-proBNP elevations.

**Table 3:** Association of patient characteristics with positive troponin—univariate and multivariate regression analyses.

Variable	Positive troponin (n, %)	Univariate regression analysis		Multivariate regression analysis	
		Odds ratio [confidence interval]	*p* value	Odds ratio [confidence interval]	*p* value
**Age (> 60 years)**	56 (25)	1.39 [0.87 to 2.25]	0.187	1.13 [0.59 to 2.16]	0.705
**Sex (female)**	32 (13)	0.50 [0.31 to 0.80]	0.004*	0.91 [0.47 to 1.77]	0.909
**SBP (> 130 mm Hg)**	18 (20)	1.18 [0.66 to 2.10]	0.573	1.24 [0.62 to 2.49]	0.550
**Heart rate (> 100 bpm)**	6 (22)	1.38 [0.54 to 3.54]	0.504	0.60 [0.19 to 1.90]	0.385
**Cardiotoxicity**	12 (40)	3.11 [1.44 to 6.73]	0.004*	1.93 [0.76 to 4.93]	0.300
**Echocardiographic findings**					
LVEF (< 50%)	53 (46)	8.03 [4.85 to 13.30]	<0.001*	8.42 [4.63 to 15.28]	<0.001*
Diastolic dysfunction	28 (29)	2.18 [1.30 to 3.66]	0.003*	1.83 [0.95 to 3.53]	0.070*
GLS (> −20)	24 (33)	2.60 [1.49 to 4.53]	0.001*	1.95 [0.97 to 3.95]	0.061
**Comorbidities**					
Coronary artery disease	26 (31)	2.26 [1.32 to 3.88]	0.003*	1.36 [0.77 to 2.43]	0.293
Hypertension	50 (24)	1.29 [0.93 to 1.90]	0.126	1.19 [0.81 to 1.76]	0.371
Atrial fibrillation	21 (36)	2.84 [1.57 to 5.15]	0.001*	1.56 [0.74 to 3.24]	0.241
**Oncological disease**					
Breast cancer	114 (12)	0.04 [0.29 to 0.98]	0.040*	0.44 [0.17 to 0.15]	0.094
Malignant melanoma	12 (9)	0.37 [0.19 to 0.71]	0.003*	0.70 [0.27 to 1.81]	0.458
Leukemia	16 (29)	2.26 [1.21 to 4.23]	0.011*	1.15 [0.48 to 2.77]	0.750
Lymphoma	8 (22)	1.32 [0.58 to 3.00]	0.511	0.57 [0.18 to 1.84]	0.347
**Antineoplastic treatment**					
Anthracycline	27 (24)	1.50 [0.90 to 2.52]	0.120	1.24 [0.55 to 2.81]	0.607
Alkylation	34 (24)	1.67 [1.03 to 2.72]	0.040*	1.21 [0.57 to 2.56]	0.616
Taxane	18 (23)	1.08 [0.90 to 1.30]	0.202	0.19 [0.75 to 1.87]	0.459
PD1 inhibitor	12 (117)	1.05 [0.98 to 1.13]	0.144	0.82 [0.35 to 1.93]	0.643
BRAF/MEK inhibitor	4 (15)	0.77 [0.26 to 2.30]	0.645	1.82 [0.47 to 7.09]	0.388

*p < 0.05.

bpm, beats per minute; BRAF, B-Rapidly growing fibrosarcoma; GLS, global longitudinal strain; LVEF, left ventricular ejection fraction; MEK, mitogen-activated protein kinase; n, number; SBP, systolic blood pressure; PD1, programmed cell death protein 1.

**Table 4:** Association of patient characteristics with positive NT-proBNP—univariate and multivariate regression analyses.

Variable	Positive NT-proBNP (n, %)	Univariate regression analysis		Multivariate regression analysis	
		Odds ratio [confidence interval]	p-value	Odds ratio [confidence interval]	p-value
**Age (> 60 years)**	97 (35)	1.25 [0.85 to 1.84]	0.252	0.93 [0.57 to 1.70]	0.950
**Sex (female)**	68 (28)	0.64 [0.44 to 0.93]	0.021*	1.11 [0.63 to 1.98]	0.713
**SBP (> 130 mmHg)**	34 (38)	1.34 [0.83 to 2.16]	0.229	1.58 [0.85 to 2.92]	0.148
**Heart rate (> 100 bpm)**	10 (38)	1.30 [0.58 to 2.93]	0.530	0.51 [0.15 to 1,76]	0.289
**Cardiotoxicity**	21 (70)	4.95 [2.21 to 11.10]	<0.001*	3.55 [1.29 to 9.80]	0.014*
**Echocardiographic findings**					
LVEF (< 50%)	86 (75)	11.62 [7.13 to 18.94]	<0.001*	20.84 [10.67 to 40.70]	<0,001*
Diastolic dysfunction	42 (43)	1.73 [1.10 to 2.73]	0.018*	1.06 [0.57 to 1.96]	0.865
GLS (> −20)	43 (58)	3.53 [2.12 to 5.87]	<0.001*	2.58 [1.32 to 5.02]	0.005*
**Comorbidities**					
Coronary artery disease	47 (56)	1.03 [1.01 to 1.06]	0.009*	1.48 [0.82 to 2.65]	0.191
Hypertension	86 (42)	1.03 [1.01 to 1.05]	0.008*	1.39 [0.94 to 2.06]	0.095
Atrial fibrillation	34 (58)	1.03 [1.01 to 1.05]	0.009*	0.97 [0.47 to 1.97]	0.927
**Oncological disease**					
Breast cancer	35 (30)	0.83 [0.53 to 1.30]	0.407	0.49 [0.23 to 1.08]	0.078
Malignant melanoma	22 (17)	0.32 [0.20 to 0.54]	<0.001*	0.57 [0.27 to 1.23]	0.152
Leukemia	24 (44)	1.69 [0.96 to 3.00]	0.071*	0.79 [0.34 to 1.84]	0.588
Lymphoma	13 (36)	1.17 [0.58 to 2.38]	0.659	0.53 [0.18 to 1.53]	0.240
**Antineoplastic treatment**					
Anthracycline	51 (45)	1.03 [1.01 to 10.05]	0.008*	1.42 [0.68 to 27.9]	0.348
Alkylation	59 (42)	1.03 [1.01 to 1.05]	0.008*	0.68 [0.34 to 1.36]	0.275
Taxane	37 (46)	1.03 [1.01 to 1.05]	0.008*	1.05 [0.70 to 1.57]	0.828
PD1 inhibitor	25 (24)	1.03 [1.01 to 1.05]	0.007*	0.50 [0.27 to 0.95]	0.033*
BRAF/MEK inhibitor	5 (19)	0.45 [0.17 to 1.21]	<0.001*	0.78 [0.22 to 2.76]	0.695

*p < 0.05.

bpm, beats per minute; BRAF, B-Rapidly growing fibrosarcoma; GLS, global longitudinal strain; LVEF, left ventricular ejection fraction; MEK, mitogen-activated protein kinase; n, number; NT-proBNP, N-terminal pro B-type natriuretic peptide; SBP, systolic blood pressure; PD1, programmed cell death protein 1.

Patients with previously known or newly diagnosed cardiac comorbidities showed positive troponin and NT-proBNP values more frequently. Univariate analysis revealed significantly higher rates of positive NT-proBNP in patients with coronary artery disease (OR, 1.03 [1.01 to 1.06], *p* = 0.009), arterial hypertension (OR, 1.03 [1.01 to 1.05], *p* = 0.008) and atrial fibrillation (OR, 1.03 [1.01 to 1.05], *p* = 0.009). Multivariate analysis showed no significant association. Moreover, patients with coronary artery disease (OR, 2.26 [1.32 to 3.88], *p* = 0.003) and atrial fibrillation (OR, 2.84 [1.57 to 5.15], *p* = 0.001) had higher rates of positive troponin in univariate analysis.

Moreover, univariate analysis showed significantly higher rates of positive troponin in patients treated with alkylating agents (OR, 1.67 [1.03 to 2.72], *p* = 0.040). In addition, univariate analysis revealed higher rates of positive troponin in patients treated with anthracyclines, alkylating agents, taxane, PD1 inhibitors, and BRAF/MEK inhibitors.

### Association of Echocardiographic Findings and Biomarkers

**Figure 1:**
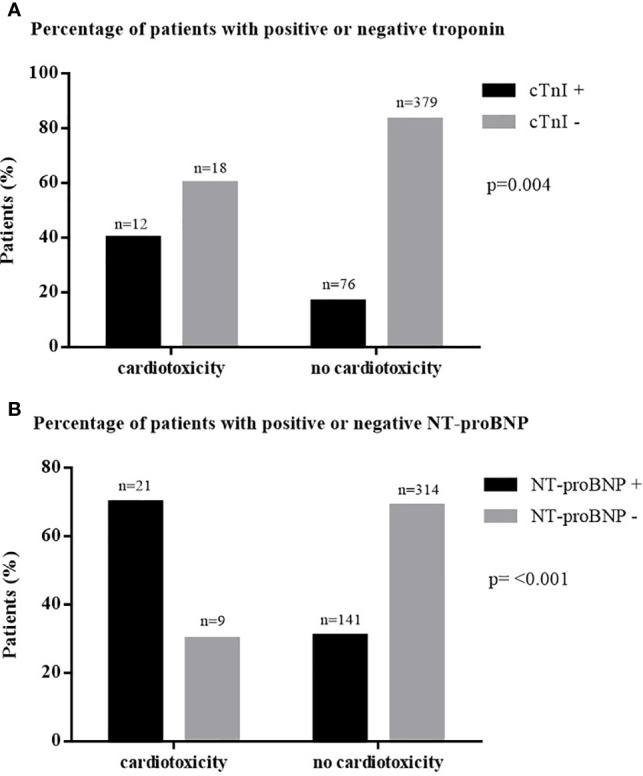
Percentage of patients with elevated biomarkers in cancer therapy-induced cardiotoxicity. **(A)** Percentage of patients with positive or negative troponin. **(B)** Percentage of patients with positive or negative NT-proBNP. cTnI, cardiac troponin I; n, number; NT-proBNP, N-terminal pro B-type natriuretic peptide.

In cross-sectional data, Spearman rank correlation analysis showed an association between elevated NT-proBNP and a reduction in LVEF (*p* < 0.001, *r* = 0.406). Furthermore, an increased cTnI correlated significantly with LVEF reduction (*p* < 0.001, *r* = 0.234). The percentage of patients with positive cTnI and NT-proBNP was higher in patients with EF <40% and EF between 40% and 50% than in patients with EF > 50% (**Figure 1**).

Moreover, univariate binary logistic regression showed that the presence of diastolic dysfunction was associated with positive troponin (OR, 2.18 [1.3 to 3.66], *p* = 0.003) and NT-proBNP (OR, 1.73 [1.10 to 2.73], *p* = 0.018).

GLS was performed in 210 patients. Seventy-four of them showed pathological strain values (defined as GLS > −20). Univariate and multivariate analyses showed a significant association between elevated NT-pro-BNP and pathological strain measurements (OR, 2.58 [1.32 to 5.02], *p* = 0.005). Moreover, univariate analysis showed an association between increased troponin and pathological GLS (OR, 2.60 [1.49 to 4.53], *p* = 0.001).

**Figure 2:**
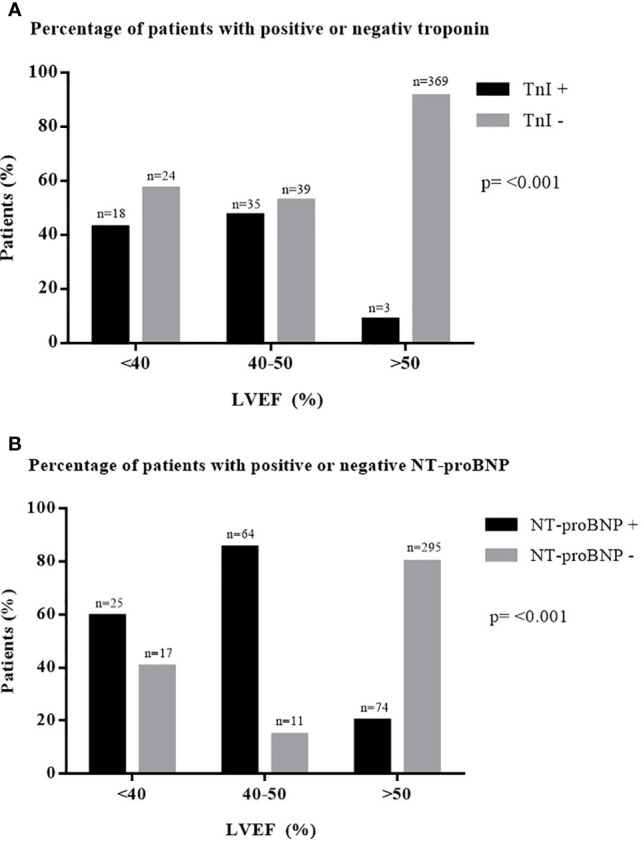
Percentage of patients with elevated biomarkers in different degrees of LVEF. **(A)** Percentage of patients with positive or negative troponin. **(B)** Percentage of patients with positive or negative NT-proBNP. cTnI, cardiac troponin I; LVEF, left ventricular ejection fraction; n, number; NT-proBNP, N-terminal pro B-type natriuretic peptide.

N = 30 patients presented with chemotherapy-induced cardiomyopathy, defined as a significant LVEF decrease (> 10%) to a value below 50% (Zamorano, 2016). The number of patients with increased cTnI and NT-proBNP was significantly higher in patients with chemotherapy-induced cardiomyopathy than in patients without cardiotoxicity (**Figure 2**).

### Sensitivity and Specificity Testing

Troponin showed a high specificity of 95.47% with a low sensitivity of 13.64% for the detection of chemotherapy-related cardiotoxicity. Likewise, NT-proBNP showed a high specificity of 92.21% with a low sensitivity of 12.06% for the detection of chemotherapy-related cardiotoxicity.

### Follow-Up Investigations

#### Changes in Biomarkers and LVEF in the Total Cohort

To investigate a potential association between changes in biomarker values and the changes in LVEF between baseline and follow-up measurements, a Spearman rank correlation analysis was performed. The analysis showed a correlation between changes in cTnI and changes in LVEF (*p* = 0.012, *r* = 0.231) and between changes in NT-proBNP and changes in LVEF (*p* = 0.001, *r* = 0.300).

#### Changes in Biomarkers and LVEF in Subgroup Analysis

A total of n = 42 patients treated with melanoma therapy received serial measurements of LVEF and cardiac biomarkers. Similar to the total cohort, melanoma patients showed a moderate correlation between the change in cTnI and the change in LVEF during follow-up (*p* = 0.002, *r* = 0.456), whereas no significant correlation could be found between the change in NT-proBNP and the change in LVEF (*p* = 0.555, *r* = 0.094). N = 42 patients with breast cancer therapy received a follow-up investigation. In this subgroup, a correlation for the change in NT-proBNP with the change in LVEF could be detected (*p* = 0.003, r = 0.449). There was no significant correlation between changes in cTnI and changes in NT-proBNP in breast cancer patients (*p* = 0.213, *r* = 0.195).

## Discussion

Despite their therapeutic efficacy, the possible adverse cardiovascular side effects of antineoplastic treatments are increasingly reported (Raschi and De Ponti, 2012). The impairment of LVEF is the most common manifestation of cancer therapy-induced cardiotoxicity (Zamorano et al., 2016). The early detection of cancer therapy-related myocardial dysfunction is one of the major challenges in the field of cardio-oncology, and it enables treating physicians to modify the oncological regime to a less cardiotoxic schedule (Rassaf and Totzeck, 2018). Moreover, timely diagnosis of cardiotoxicity can enable an early initialization of cardioprotective agents (Cardinale et al., 2006; Cardinale et al., 2008; Cardinale et al., 2013; Chang et al., 2017a; Chang et al., 2017b; Cardinale et al., 2018). This sensitive topic is especially important in patients with neoplastic diseases, in whom the occurrence of myocardial dysfunction restricts the therapeutic options, thereby negatively affecting the outcome (Cardinale et al., 2008).

Cardiac biomarkers are a cornerstone for the diagnosis of acute and chronic cardiovascular diseases (Vavuranakis et al., 2012). Many previous studies have investigated the diagnostic and prognostic value of cTnI and NT-proBNP in the risk assessment of acute ischemia and heart failure (Rassaf and Totzeck, 2018). To date, there is inconsistent evidence regarding the benefits of using biomarkers for screening for cardiotoxicity in tumor patients. Few studies failed to detect important changes in cTnI and NT-proBNP during and following cancer treatment (Mathew et al., 2001; Vogelsang et al., 2008; Pavo et al., 2015). Other studies have shown an association of early and persistent cTnI elevations with cardiovascular events in cancer patients (Cardinale et al., 2000; Cardinale et al., 2004; Specchia et al., 2005).

Based on the present cross-sectional study, the evaluation of the heart failure marker NT-proBNP may be a diagnostic tool for the detection of both systolic and diastolic heart failure in tumor patients. NT-proBNP is known for its high prognostic value for the detection of impaired cardiac function in patients with ischemic and dilated cardiomyopathy (Vavuranakis et al., 2012). Our results are in concordance with most of the recent studies, which have elucidated the correlation between increased NT-proBNP and abnormal myocardial function in patients treated with cytotoxic agents in general, especially in breast cancer patients treated with anthracyclines (Bauch et al., 1992; Suzuki et al., 1998; Nousiainen et al., 2002; Sandri et al., 2005; Soker and Kervancioglu, 2005; Germanakis et al., 2006; Aggarwal et al., 2007). Nevertheless, 43% of the patients with a reduced LVEF <50% showed no increase in the NT-proBNP values.

Troponin, on the other hand, appears to be particularly useful for the detection of acute cardiotoxicity. In the overall cohort and in the subgroup of melanoma patients who received a short-term follow-up examination, a change in cTnI level correlated with a change in LVEF. A total of n = 98 of the n = 485 patients had positive troponin, and n = 63 of these were at the low end of the detectable range. cTnI is a parameter of myocardial damage, and previous studies have shown that low-level increases in cTnI following acute and chronic ischemia are associated with a higher risk of recurrent cardiovascular events and mortality (Galvani et al., 1997; Roos et al., 2017). Furthermore, for high-dose chemotherapy and immune checkpoint inhibitor therapy, a correlation between prolonged troponin elevation and increased risk for cardiovascular events was demonstrated (Cardinale et al., 2000; Cardinale et al., 2004; Mahmood et al., 2018; Michel et al., 2018). These troponin elevations may be a sign of myocardial damage and are an indication for more stringent follow-up visits.

In the present study, cardiac comorbidities, such as coronary heart disease, arterial hypertension, and atrial fibrillation, were associated with an increased incidence of cardiac biomarkers. Some authors speculate about a hypothesis in which antineoplastic treatment leads to cardiotoxic effects, while increased cardiovascular risk factors synergistically contribute to an elevated risk of cardiovascular diseases (Jones et al., 2007; Gunaldi et al., 2016).

Our investigation of specificity and sensitivity showed a high specificity and a low sensitivity of cTnI and NT-proBNP for the detection of cancer therapy-related cardiotoxicity. Our data demonstrate that positive TnI and NT-proBNP predict cardiotoxicity with high probability. However, due to the low sensitivity, negative values cannot rule out cancer therapy-induced cardiotoxicity. These results are in accordance with the current evidence that an assessment of NT-proBNP should be performed particularly for the evaluation of symptomatic patients with suspected heart failure and is less useful for the investigation of asymptomatic high-risk patients (Ponikowski et al., 2016).

### Limitations

The study had a short follow-up period of 1–6 months. Due to the retrospective design of the study, not all included patients received follow-up examinations, and the timepoints of the follow-up examinations differed between the patients, leading to increased comparability. However, the cohort analyzed here represents a population in which biomarker assessment remains to be studied in detail.

The included patients show heterogeneous characteristics, e.g., cancer entity and the type of cancer therapy and comorbidities. Cumulative analysis does not facilitate the establishment of generalized clinical recommendations on the use of biomarkers. However, we are convinced that our study will serve to better understand biomarker elevation in the real-world patient collective.

The sample size of our study may not be sufficient to make significant statements about the influence of different tumor therapies on the dynamic increase in biomarkers. However, patients treated with anthracyclines, alkylation, taxanes, and PD1 inhibitors appear to have increased NT-pro-BNP levels more often. Due to the short follow-up period, most patients received one or two chemotherapy cycles. Therefore, the cumulative dose of chemotherapy, e.g., anthracyclines, was low. Further randomized studies with longer follow-up periods will be necessary to further evaluate the benefits of cardiac biomarker evaluation in different tumor groups.

Moreover, some of the included patients had previous cardiac diseases. Comorbidities such as atrial fibrillation can also lead to an increase in cardiac biomarkers. A total of 10 patients had permanent atrial fibrillation. In these patients, elevated NT-proBNP values can also be partly caused by cardiac arrhythmias.

The number of patients with serial measurements was only 116 patients. Prospective studies with a large number of tumor patients are necessary to validate the results and to investigate various subgroups.

## Conclusion

Our data suggest that cardiac biomarkers play an important role in the detection of cancer therapy-induced cardiotoxicity. However, despite moderate correlations between both NT-proBNP and cTnI and reduced LVEF measurements, neither baseline value nor follow-up investigations of cardiac biomarkers can safely replace advanced monitoring in patients undergoing cancer therapy. In addition to the assessment of cardiac biomarkers, their combination with additional investigations, including anamnesis, clinical examination, electrocardiography, and transthoracic echocardiography with strain analysis, should still be regarded as the preferred screening method for the detection of early myocardial damage in high-risk patients.

## Data Availability Statement

The authors confirm that the data supporting the findings of this study are available within the article.

## Ethics Statement

The studies involving human participants were reviewed and approved by ethics committee of the University of Duisburg-Essen. The patients/participants provided their written informed consent to participate in this study.

## Author Contributions

(I) Conception and design: LH, MT. (II) Administrative support: TR, MT. (III) Provision of study materials or patients: LH, SM, R-IM, JP, AR. (IV) Collection and assembly of data: LH, SM, R-IM, JP, AR. (V) Data analysis and interpretation: LH, MT, AM. (VI) Manuscript writing: All authors. (VII) Final approval of manuscript: All authors.

## Funding

TR was funded by the German Research Foundation (Deutsche Forschungsgemeinschaft, DFG) (RA 964/12-2). SM was funded by the IFORES research grant from the Medical Faculty, University Duisburg-Essen, Germany.

## Conflict of Interest

The authors declare that the research was conducted in the absence of any commercial or financial relationships that could be construed as a potential conflict of interest.

## Abbreviations

bpm, beats per minute; BRAF, B-Rapidly growing fibrosarcoma; CCS, Canadian Cardiovascular Society; COPD, chronic obstructive pulmonary disease; cTnI, cardiac troponin I; HER2, human epidermal growth factor receptor 2; LVEF, left ventricular ejection fraction; MEK, mitogen-activated protein kinase; NT-proBNP, N-terminal pro B-type natriuretic peptide; NYHA, New York Heart Association; PD1, programmed cell death protein 1.
